# Alcohol and Aldehyde Dehydrogenases Contribute to Sex-Related Differences in Clearance of Zolpidem in Rats

**DOI:** 10.3389/fphar.2016.00260

**Published:** 2016-08-15

**Authors:** Cody J. Peer, Jonathan D. Strope, Shaunna Beedie, Ariel M. Ley, Alesia Holly, Karim Calis, Ronald Farkas, Jagan Parepally, Angela Men, Emmanuel O. Fadiran, Pamela Scott, Marjorie Jenkins, William H. Theodore, Tristan M. Sissung

**Affiliations:** ^1^Clinical Pharmacology Program, National Cancer Institute, National Institutes of Health, BethesdaMD, USA; ^2^Molecular Pharmacology Program, National Cancer Institute, National Institutes of Health, BethesdaMD, USA; ^3^Office of Medical Policy, Center for Drug Evaluation and Research, Food and Drug Administration, Silver SpringMD, USA; ^4^Office of New Drugs, Division of Neurology Products, Center for Drug Evaluation and Research, Food and Drug Administration, Silver SpringMD, USA; ^5^Office of Clinical Pharmacology, Office of Translational Sciences, Center for Drug Evaluation and Research, Food and Drug Administration, Silver SpringMD, USA; ^6^Office of Women’s Health, Office of the Commissioner, Food and Drug Administration, Silver SpringMD, USA; ^7^Clinical Epilepsy Section, National Institute of Neurological Disorders and Stroke, National Institutes of Health, BethesdaMD, USA

**Keywords:** zolpidem, drug metabolism, pharmacokinetics, testosterone

## Abstract

**Objectives:** The recommended zolpidem starting dose was lowered in females (5 mg vs. 10 mg) since side effects were more frequent and severe than those of males; the mechanism underlying sex differences in pharmacokinetics (PK) is unknown. We hypothesized that such differences were caused by known sex-related variability in alcohol dehydrogenase (ADH) expression.

**Methods:** Male, female, and castrated male rats were administered 2.6 mg/kg zolpidem, ± disulfiram (ADH/ALDH pathway inhibitor) to compare PK changes induced by sex and gonadal hormones. PK analyses were conducted in rat plasma and rat brain.

**Key findings:** Sex differences in PK were evident: females had a higher *C*_MAX_ (112.4 vs. 68.1 ug/L) and AUC (537.8 vs. 231.8 h^∗^ug/L) than uncastrated males. Castration induced an earlier *T*_MAX_ (0.25 vs. 1 h), greater *C*_MAX_ (109.1 vs. 68.1 ug/L), and a corresponding AUC increase (339.7 vs. 231.8 h^∗^ug/L). Administration of disulfiram caused more drastic *C*_MAX_ and *T*_MAX_ changes in male vs. female rats that mirrored the effects of castration on first-pass metabolism, suggesting that the observed PK differences may be caused by ADH/ALDH expression. Brain concentrations paralleled plasma concentrations.

**Conclusion:** These findings indicate that sex differences in zolpidem PK are influenced by variation in the expression of ADH/ALDH due to gonadal androgens.

## Introduction

Zolpidem is a gamma-aminobutyric acid (GABA) agonist that is indicated for the treatment of insomnia characterized by difficulties with sleep initiation. Sex-specific differences in pharmacokinetics (PK) have been observed in which females have greater exposure than males and have increased probabilities of experiencing undesired, persistent pharmacological effect after waking (typically drowsiness) (Greenblatt et al., 2000, 2014a; Verster et al., 2014). To address sex-related adverse events, the FDA reduced the recommended initial dose by half in females (Food and Drug Administration [FDA], 2008).

Zolpidem is hydroxylated by CYP3A4, rapidly oxidized to an aldehyde by alcohol dehydrogenases (ADHs), and finally converted into a carboxylic acid by aldehyde dehydrogenases (ALDHs). The major circulating metabolite is zolpidem phenyl 4-carboxylic acid (ZPCA; 72–86%), with zolpidem 6-carboxylic acid (ZCA) making up roughly ∼10% of the administered dose (Gillet, 1991; Pichard et al., 1995). It is currently unknown which ADH/ALDH enzyme classes are responsible for the metabolism of zolpidem, and whether gastric ADH/ALDH pathways contribute to zolpidem disposition. Nevertheless, along with certain cytochromes P450, such as CYP3A that acts synergistically with other enzymes in the gastric mucosa of humans and animals (Yoon et al., 2011), the ADH/ALDH enzyme pathway is likely the major contributor to the bioavailability and elimination of zolpidem.

CYP3A4 activity is greater in females and is therefore unlikely to result in slower metabolism in women (Wolbold et al., 2003). However, it is well known that ADH/ALDH expression in the gastrointestinal (GI) tract is much lower in females vs. males, likely due to differences in androgens (**Table 1**). Low ADH activity would be expected to slow CYP3A4 zolpidem metabolism due to inefficient removal of the products of CYP3A4-mediated zolpidem metabolism, based on Le Chatelier’s principle. Sex hormone concentration differences are also responsible for variability in ADH expression in aging, which is consistent with the observed reduction in zolpidem clearance in older individuals (Greenblatt et al., 2014b). Similar sexual- and age-dimorphic Adh expression profiles have also been observed in the GI tracts and livers of rats (Estonius et al., 1993; Aasmoe and Aarbakke, 1999; Westerlund et al., 2007).

**Table 1:** Alcohol dehydrogenase (Adh) Class 1-V and Aldh isozymes in humans and rats.

Enzyme class	Isozymes in humans	Human expression sites	Rat expression sites	Sexually dimorphic expression M vs. F	Metabolic parameter	Reference
**ADH**					**Ethanol Km (mM), Vmax (min^-1^)**	
Class I	*ADH1A ADH1B^∗^1, ADHB^∗^2, ADH1B^∗^3, ADH1C^∗^1, ADH1C^∗^2*	Liver (all) and stomach (ADH1C^∗^1)	Duodenum, colon, rectum, liver, kidney, stomach, greatest expression in the above tissues vs. other ADH classes.	Higher in female rats’ liver Lower in female rats’ gastrointestinal tract	4.0, 30 0.05, 4 0.9, 350 40.0, 300 1.0, 90 0.6, 40	Estonius et al., 1993; Aasmoe and Aarbakke, 1999; Westerlund et al., 2007
ADH2	*ADH4*	Liver	Liver, kidney, stomach, duodenum	Lower in female rats’ liver	30, 20	Estonius et al., 1993; Aasmoe and Aarbakke, 1999
ADH3	*ADH5*	Most tissues	Tongue, esophagus, stomach, duodenum, jejunum, Ileum, colon, rectum, liver, kidney, stomach	None	>1000, 100	Estonius et al., 1993; Westerlund et al., 2007
ADH4	*ADH7*	Gastric mucosa	Tongue, esophagus, stomach	?	30, 1800	Westerlund et al., 2007
ADH5	ADH6		Liver, stomach	?	?. ?	
**ALDH (9 classes, but 3 major in rats)**					**Disulfiram IC50 of acetaldehyde metabolism (uM)**	Quertemont, 2004
ALDH1 (RalDH)	None	Cytosolic (high *K*_m_ for acetaldehyde)	Tongue, esophagus, stomach, duodenum, jejunum, not detectible in liver	?	Human 0.15 Rat0.10	Keung and Vallee, 1993; Chen et al., 1996; Westerlund et al., 2007; Koppaka et al., 2012
ALDH2	None	Mitochondria (Low *K*_m_ for acetaldehyde)	Liver	?	Human 1.45 Rat >20	Keung and Vallee, 1993; Koppaka et al., 2012
ALDH3	Constitutive, xenobiotic inducible		GI tract	?	?	Lindahl, 1992; Xie et al., 1996

ADH expression is greatest in the liver while smaller amounts have been observed in the GI tract and the kidney (Estonius et al., 1993; Aasmoe and Aarbakke, 1999; Westerlund et al., 2007). The ALDH inhibitor, disulfiram, slows ADH metabolism by irreversibly inhibiting the elimination of aldehydes formed by ALDH (IC_50_ = 0.15 uM for ALDH1; IC_50_ = 1.45 uM for ALDH2) (Koppaka et al., 2012) and therefore shifts the alcohol-aldehyde equilibrium toward hydroxides (Brien et al., 1978; Cederbaum, 2012). Without ADH/ALDH to further metabolize hydroxyl metabolites, a buildup of these could be compensated by reduced production by CYPs, possibly due to mechanism-based inhibition (Polasek et al., 2010). However, a potential zolpidem/disulfiram interaction has not been studied. We therefore hypothesized that sexual dimorphism in the expression of ADH and ALDH in males and females is responsible for the observed sex differences in zolpidem exposure, and that disulfiram treatment would therefore have similar effects as castration via bypass of gastric ADH/ALDH metabolism. The preclinical pilot study described here details the PK analysis of zolpidem in Sprague–Dawley rats in order to provide a preliminary understanding of the observed clinical differences in zolpidem PK and pharmacodynamics (PD) between males and females.

## Materials and Methods

### Materials

Zolpidem free base (≥98% purity by HPLC) was purchased from Sigma–Aldrich (St. Louis, MO, USA) through the NIH Pharmacy. Zolpidem phenyl 4-carboxylic acid (ZPCA), ZCA, and [H^2^]_6_-zolpidem (D6-zolpidem) were purchased from Toronto Research Chemicals (Toronto, ON, Canada). Stock solutions were prepared in DMSO (Sigma–Aldrich) that was subsequently diluted in a 1% sucrose (aq) solution for oral gavage. Male and female Sprague–Dawley rats were obtained from Charles River Labs (Germantown, MD, USA). Disulfiram was purchased from Sigma–Aldrich (St. Louis, MO, USA) as European Pharmacopeia grade reference standard and was formulated for intraperitoneal injection in a 1% suspension of carboxy methylcellulose (CMC), purchased from Sigma–Aldrich (St. Louis, MO, USA), as previously described (Sharkawi, 1980).

### Study Design

Rats were separated into 5 treatment groups, each receiving 2.6 mg/kg zolpidem: (1) Group 1: uncastrated males receiving CMC vehicle control (MC); (2) Group 2: uncastrated males receiving disulfiram (MD); (3) Group 3: females receiving disulfiram (FD); (4) Group 4: females receiving CMC vehicle control (FC); and (5) Group 5: castrated males receiving CMC vehicle control (cMC). Disulfiram was administered intraperitoneally at a dose of 100 mg/kg approximately 16 h before the middle of each zolpidem time point in order to ensure Aldh inhibition was maximal (Gessner and Gessner, 1992). For example, rats corresponding to the 8 h time point were administered disulfiram 12 h prior to zolpidem administration (mid-point of the 8-h time is 4 h, thus 12 h prior to dose is 16 h) whereas rats corresponding to the 1 h time point were treated 15.5 h prior to zolpidem administration. All rats were given food and water ad libidum. Zolpidem was administered as an oral gavage (volume 2 mL) at a dose of 2.6 mg/kg as had been used previously (Garrigou-Gadenne et al., 1989). Male rats in Group 5 (cMC) were castrated according to an NIH Animal Care and Use Committee (ACUC) approved procedure (Dulisch, 1976). Briefly, castration surgeries were preformed through the abdominal wall. To initiate and maintain the plane of anesthesia, 2.5% vaporized isoflurane was inhaled with a 1.5 L/min flow rate. Excision of the entire testicle and epididymis was accomplished by suturing spermatic vessels and vas deferens closed. The peritoneum was closed with 4–0 absorbable sutures and the outer abdominal skin with surgical clips. Marcaine drops were used as a nerve block and buprenorphine as an analgesic. During recovery, animals were kept warm and watched until able to walk. They were then returned to their cages and per protocol, were checked every day after surgery for 5 days. After 10 days, surgical clips were removed. All castrated males were treated with zolpidem 14 days after castration since serum testosterone concentrations approach levels observed in females after that time, and a previous work demonstrated Adh/Aldh differences in rats that were only castrated for 7 days (Aasmoe and Aarbakke, 1999; Christoffersen et al., 2006). All animal care and maintenance was in accordance with NIH ACUC guidelines.

### PK Sampling and Sample Bioanalysis

To examine the PK profile of zolpidem following oral gavage, blood samples were collected in heparinized tubes via cardiac puncture, and immediately placed on ice. Carbon dioxide asphyxiation was conducted to ensure euthanasia and brains were then harvested from each rat. Samples (plasma and whole brain) were obtained at 5 min, 15 min, 30 min, 1 h, 2 h, 3 h, and 8 h post gavage. Each time point was performed in three rats within each group (seven time points in triplicate = 21 rats per group). Immediately after collection, blood samples were centrifuged for 5 min at 1200 ×*g*. The plasma supernatant was then immediately transferred to a cryovial and stored at -80°C until the time of bioanalysis. Brains were snap-frozen and stored until needed, when they were thawed and homogenized for bioanalysis. Plasma and brain concentrations of zolpidem and its two major carboxylic acid metabolites ZPCA and ZCA were quantitatively measured using a validated HPLC with tandem mass spectrometric detection (HPLC-MS/MS) method with a calibration range of 0.5–1,000 ug/L (ng/mL). Briefly, 100 uL of rat plasma was spiked with ^2^[H]_6_-zolpidem (internal standard), acetonitrile was then added to precipitate proteins. The acetonitrile extract was dried and the residue reconstituted. Zolpidem, ZPCA, and ZCA had chromatographic retention times of 4.8min, 3.6min, and 4.1 min, respectively; the deuterated internal standard (^2^[H]_6_-zolpidem) eluted at the same time as unlabeled zolpidem The assay was validated per FDA guidelines, with accuracy and precision of calibration standards and quality control standards less than 15% (Food and Drug Administration [FDA], 2015).

### Non-compartmental Analysis

A naïve-pooled, sparse non-compartmental (model-independent) approach was used to calculate plasma and brain PK parameters of zolpidem and the two metabolites, treating all data as originating in one “average” rat, using Phoenix WinNonlin v6.4 (Certara Pharsight Corporation, Cary, NC, USA). Any plasma or brain concentration measured below the LLOQ (0.5 ug/L) was excluded from analyses. The maximum plasma concentration (*C*_MAX_) and time to *C*_MAX_ (*T*_MAX_) were recorded as the mean (*n* = 3 per group) of observed values; the area under the plasma concentration vs. time curve (AUC_LAST_) was calculated using the linear trapezoidal rule as a linear combination of the mean concentration values using observable times up to 8 h. The standard error (SE) of the average *C*_MAX_ value in each treatment group was calculated as the sample standard error of the concentration values at the observed *T*_MAX_. The SE of the mean AUC_LAST_ estimate from destructive sampling was calculated according to the method of Nedelman and Jia (1998). Bailer’s method *Z*-tests were used to calculate the statistical differences in AUC between groups of rats using Microsoft Excel^®^ (Bailer, 1988). Graphs were prepared using GraphPad Prism, v6.01 (GraphPad Software, San Diego, CA, USA) as well as all statistical analyses (except Bailer’s), where a two-sided *p* < 0.05 was considered to be statistically significant.

### Statistical Considerations

Comparisons between PK parameters (except AUC, which was calculated with Bailer’s analysis) were conducted with the Student’s *t*-test. Several comparisons were conducted with only *n* = 3 rats in each group; therefore, individual PK parameters have low power to detect differences between rats and comparisons of such data will be reported as the mean and 95% CI.

## Results

### Comparison of Zolpidem PK by Sex and Castration Status

**FIGURE 1:**
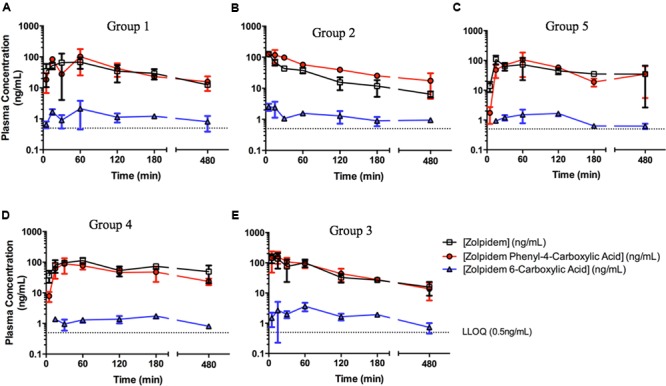
**Zolpidem plasma concentration vs. time profiles in **(A)** Group 1: uncastrated males + vehicle (1% CMC, i.p.), **(B)** Group 2: uncastrated males + disulfiram (suspended in 1% CMC, i.p.), **(C)** Group 5: castrated males + vehicle, **(D)** Group 4: females + vehicle, and **(E)** Group 3: females + disulfiram.** Zolpidem (open squares), the major metabolite zolpidem phenyl 4-carboxylic acid (red circles), and the minor metabolite zolpidem 6-carboxylic acid (ZCA) (blue triangles) were measured in rat plasma at varying time points post oral gavage of 2.6 mg/kg either with the ADH/ALDH inhibitor disulfiram, or its vehicle (CMC).

**FIGURE 2:**
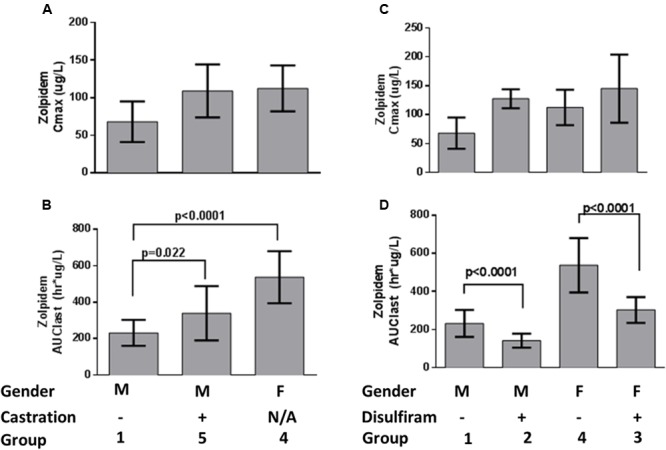
**Zolpidem plasma **(A)***C*_max_ and **(B)** AUC_last_ by sex and castration status, and **(C)***C*_max_ and **(D)** AUC_last_ by sex and disulfiram status.** Data are represented by bar graphs depicting the mean ± the standard error of the mean (SEM), and *p*-values were determined by Bailer’s *Z*-test.

The concentration-time profiles for zolpidem, ZPCA and ZCA from each treatment group are depicted in **Figure 1**. There was relatively low response variability at most time points, however, eight individual data points (i.e., individual samples from eight rats) were excluded from analyses either due to noted errors in gavage or blood draw technique (7/8), or the resulting plasma concentration from bioanalysis being approximately 10 SDs above the mean (1/8). The PK of zolpidem in males and females treated with vehicle (1% CMC) were compared to demonstrate the existence of sexual dimorphism in zolpidem clearance our animal model. Consistent with human studies (Greenblatt et al., 2000, 2014a), female rats had a 1.7-fold higher *C*_MAX_ than males (112 [36.4–188] vs. 68.1 [0.94–135] ug/L; **Figure 2A**) and a 2.3-fold higher zolpidem AUC_LAST_ (538 [419–657] vs. 232 [172-291] h^∗^ug/L; **Figure 2B**). We therefore proposed that the gonadal testosterone secretion was, in part, responsible for this sex effect, and that this PK sex difference was likely related to differences in Adh/Aldh expression rather than Cyp3a.

To test this latter hypothesis, zolpidem PK from castrated male rats were compared to uncastrated male rats. Castrated males had 1.5-fold higher zolpidem AUC_LAST_ (340 [215–464] h^∗^ug/L) and 1.6-fold higher *C*_MAX_ (109 [21.1–197] ug/L) that was remarkably similar to females (**Figures 2A,B**) and occurred at a much earlier time point (*T*_MAX_ = 15 min) than uncastrated males and females (*T*_MAX_ = 60 min). Plasma exposure was also greater at 15 and 30 min (AUC_0-15 min_ or AUC_0-30 min_, fold change ≥1.9), further suggesting that castrated male rats were exposed to greater levels of zolpidem earlier than uncastrated males or females. Castration also induced a longer half-life (5.9 h vs. 3.3 h) compared to uncastrated males. Nevertheless, castration appeared to affect first pass metabolism more than other PK parameters, and the early rise in plasma concentration (i.e., AUC_0-30 min_ and *C*_MAX_) was solely responsible for the greater overall AUC_LAST_ observed in castrated males vs. uncastrated males.

### Comparison of Zolpidem PK with or without Disulfiram

We next determined the effect of Adh/Aldh inhibition on zolpidem metabolism by disulfiram, a potent ALDH inhibitor. Disulfiram pretreatment resulted in very rapid absorption of zolpidem (*T*_MAX_ = 5min) with a *C*_MAX_ that was 1.9-fold higher in males (128 [86.6–168] ug/L), and only slightly higher in females (145 [-1.29–291] ug/L; **Figure 2C**). This result was not unexpected as male rats have been shown to express more gastric Adh, which would be susceptible to greater inhibition than females (see **Table 1**). Within each sex receiving disulfiram, zolpidem AUC unexpectedly decreased (**Figure 2D**). This observation suggests that the effect of disulfiram on zolpidem metabolism may be dependent on the differential expression of the Adh/Aldh isoenzyme along the GI tract.

### Comparison of Zolpidem Plasma and Brain Pharmacokinetics

**FIGURE 3:**
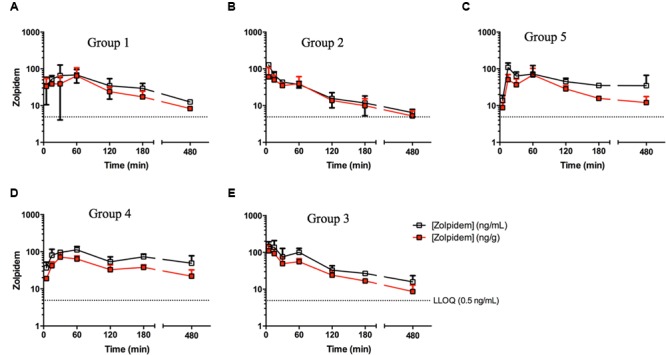
**Zolpidem plasma and brain concentration vs. time profiles in **(A)** Group 1: uncastrated males + vehicle (1% CMC, i.p.), **(B)** Group 2: uncastrated males + disulfiram (suspended in 1% CMC, i.p.), **(C)** Group 5: castrated males + vehicle, **(D)** Group 4: females + vehicle, and **(E)** Group 3: females + disulfiram.** Plasma concentrations (open squares) and brain concentrations (red squares) of zolpidem were measured in rats at varying time points post oral gavage of 2.6 mg/kg either with the ADH/ALDH inhibitor disulfiram, or its vehicle (CMC).

**FIGURE 4:**
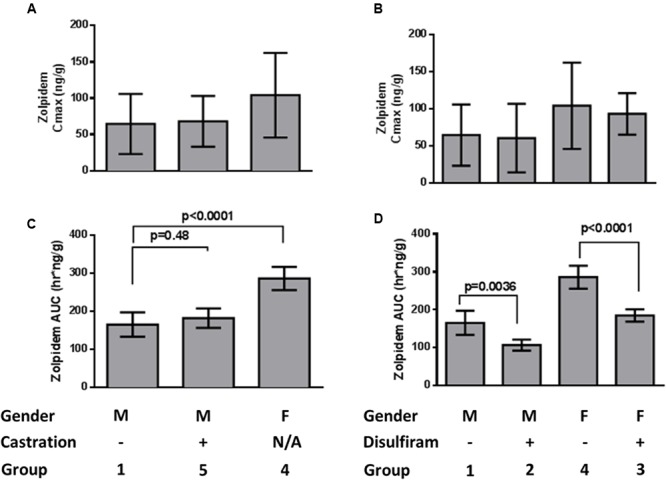
**Zolpidem brain **(A)***C*_max_ and **(B)** AUC_last_ by sex and castration status, and **(C)***C*_max_ and **(D)** AUC_last_ by sex and disulfiram status.** Data are represented by bar graphs depicting the mean ± the standard error of the mean (SEM), and *p*-values were determined by Bailer’s *Z*-test.

Brain concentrations of zolpidem strikingly paralleled the plasma profile over time, consistent with a previous report (**Figure 3**) (Garrigou-Gadenne et al., 1989). Consequently, active drug penetrates into the brain earlier (i.e., faster *T*_MAX_; 5 min vs. 1 h in males; 15 min vs. 30 min in females), and is more rapidly removed from the brain in the presence of disulfiram causing lower zolpidem brain AUC_LAST_ in females (185 [145–225] vs. 286 [211–361] h^∗^ng/g) and males (107 [70.3–143] vs. 165 [86.2–244] h^∗^ng/g; **Figure 4**). Females also had higher brain AUC (286.1 [211–361] vs. 165 [86.2–244] h^∗^ng/g) and *C*_MAX_ (104 [-40.7–249] vs. 64.5 [-38.5–167] ng/g; **Figure 4**) than males. However, brain zolpidem PK in castrated and uncastrated males were similar for *C*_MAX_ and AUC. Brain tissue exposure to ZPCA was limited, and no brain samples had measureable ZCA concentrations.

## Discussion

A sparse non-compartmental analysis demonstrated that a 2.6 mg/kg dose of zolpidem provided a *C*_MAX_ (68.1 ug/L), *T*_MAX_ (1 h) and half-life (3.3 h) in uncastrated male rats (MC) that were within reported ranges for healthy human adults given a 5 mg oral dose (**Table 2**) (Food and Drug Administration [FDA], 2008; Guo et al., 2014). Human adult *C*_MAX_, *T*_MAX_, and half-life ranged between 30 and 113 ug/L, 0.79–1.61 h, and 1.4–4.5 h, respectively, (Food and Drug Administration [FDA], 2008; Guo et al., 2014; Stockmann et al., 2014). Zolpidem AUC_LAST_ values in MC rats (Mean [95%CI]: 232 [172–291] h^∗^ug/L) matched well with a previous report in healthy human adults (234 h^∗^ug/L) (Vlase et al., 2011), but rats demonstrated a faster apparent CL/F (9 L/h/kg vs. 0.66 L/h/kg) (Olubodun et al., 2003) than humans.

**Table 2:** Pharmacokinetic (PK) Parameter Summary for Zolpidem, ZPCA, and ZCA in each Group.

Parameter	HL	Tmax	Cmax	SE_Cmax	AUCall	SE_AUCall
Units	h	h	ng/mL	ng/mL	h^∗^ng/mL	h^∗^ng/mL
**Zolpidem**					
Males	3.26	1	68.1	15.61	231.82	25.15
Males+disulfiram	2.89	0.083	127.5	9.5	141.69	12.94
Females+disulfiram	2.76	0.083	145	34	302.8	23.97
Females	8.39	1	112.35	17.65	537.77	50.36
Castrated Males	5.96	0.25	109.07	20.44	339.72	52.6
**Zolpidem Phenyl 4-Carboxylic Acid**					
Males	3.17	1	101.7	44.18	257.38	37.28
Males+disulfiram	3.54	0.083	123	5	278.2	20.94
Females+disulfiram	2.62	0.25	162.63	40.22	324.83	25.87
Females	4.22	0.5	88.25	32.75	352.1	47.29
Castrated males	6.16	1	105.77	45.07	316.84	55.49
**Zolpidem 6-Carboxylic Acid**					
Males	6.43	1	2.13	0.97	9.17	1.12
Males+disulfiram	9.2	0.083	2.52	0.455	8.69	0.062
Females+disulfiram	4.12	1	3.64	0.66	13.48	0.84
Females	6.38	3	1.74	0.164	10.31	0.591
Castrated males	6.27	2	1.65	0.176	6.92	0.432

Zolpidem phenyl 4-carboxylic acid was the predominant metabolite formed through the Cyp3a → Adh → Aldh pathway, with a metabolic ratio (metabolite:parent) for *C*_MAX_ (1.39) and AUC (1.25) that was 47-fold and 27-fold greater than that of ZCA (0.029 and 0.045, respectively). This is consistent with an *in vitro* study that demonstrated ZPCA and ZCA account for 72–86% and 10%, respectively, of urinary metabolites (Gillet, 1991; Pichard et al., 1995).

Numerous studies demonstrate that zolpidem PD effects are related to plasma concentrations (Visser et al., 2003; Verster et al., 2014), that females have higher plasma concentrations than males (Greenblatt et al., 2014a), and metabolism by CYP3A does not explain this effect since females have similar or higher CYP3A activity than males (Wolbold et al., 2003). The present data suggest that sex differences in zolpidem PK are partly a function of increased absorption, which is most likely caused by previously observed sexual differences in Adh/Aldh expression in the GI tract (Estonius et al., 1993; Aasmoe and Aarbakke, 1999; Westerlund et al., 2007). These enzymes are known to be even more sexually dimorphic in humans than in rats (fourfold vs. twofold) (Aasmoe and Aarbakke, 1999) (Parlesak et al., 2002), but this is the first study to address how Adh/Aldh variability affects zolpidem PK between sexes. It was previously demonstrated that female rats have greater activity of hepatic Adh than male rats (21.5 vs. 12.0 nmol/NADH/min/mg protein), suggesting testosterone reduces hepatic Adh expression, and therefore activity (Aasmoe and Aarbakke, 1999). This was confirmed when castrated male rats showed similar hepatic Adh specific activity to female rats (17.5 vs. 21.5 nmol/NADH/min/mg protein). The opposite was true for gastric Adh, where female and castrated male rats had lower gastric Adh specific activity (11.0 and 12.9 nmol/NADH/min/mg protein, respectively) vs. uncastrated male rats (20.5 nmol/NADH/min/mg protein) for ethanol (Aasmoe and Aarbakke, 1999). Therefore, female and castrated male rats with lower testosterone levels exhibited greater hepatic, but lower gastric, Adh activity, which manifested as less gastric metabolism (i.e., faster absorption rate, earlier *T*_MAX_, higher *C*_MAX_) and greater hepatic Adh/Aldh metabolism (i.e., more metabolite exposure). Consistent with these findings, our data showed female rats have an approximate 1.7-fold higher *C*_MAX_ and castrated males having an approximate 1.5-fold higher *C*_MAX_, and earlier *T*_MAX_ than uncastrated males. This suggests that gonadal testosterone secretion promotes gastric drug metabolism thereby acting as a barrier to drug bioavailability.

Further confirming that Adh/Aldh is responsible for sex differences in zolpidem absorption, disulfiram also caused both increased absorption rate (earlier *T*_MAX_, higher *C*_MAX_) and counterintuitively a more rapid elimination rate, which is manifested as a lower AUC in disulfiram-treated rats regardless of sex. Thus, zolpidem appears to be metabolized in part by gastric Adh/Aldh that reduces drug absorption into the systemic circulation. Similar to observations with ethanol (Ciccone and Holdcroft, 1999), a reduction in metabolism through this pathway would be expected to cause both a greater rate of absorption and increased bioavailability (Crabb et al., 1987; Greenblatt et al., 2013, 2014a). Although the specifics of *in vivo* Adh/Aldh metabolism have not been elucidated, we suggest that this is the most plausible explanation for the higher *C*_MAX_, earlier *T*_MAX_, and yet lower AUC in disulfiram-treated rats.

While previous clinical studies have reported lower systemic clearance (calculated as Dose/AUC) in females vs. males (2.7–2.8 vs. 3.9–4.0 ml/min/kg, *p* < 0.06), consistent with females having greater overall exposure, the mechanisms behind the increased exposure are not fully understood (Greenblatt et al., 2000, 2014a). It is plausible that a major source of the increased exposure in females is due to their greater absorption due to lower gastric Adh/Adh, as was observed for ethanol (Frezza et al., 1990). A lower “clearance” in females is not necessarily a factor of impaired metabolism/elimination, but rather due to increased exposure, likely from increased absorption/oral bioavailability from lower gastric ADH expression. Although the sublingual route demonstrated comparable fold-change increases in exposure (1.4-fold) in females vs. males compared to the enteral route (immediate release), this observed sex difference in exposure, and therefore clearance, in sublingual route is possibly due to ADH expression in the oral and gastric mucosa (Moreno et al., 1994; Hedberg et al., 2000). Thus, the sublingual route could still be affected by gender differences in ADH expression.

Future studies should compare zolpidem PK following IV and oral administration to study the specific contribution of gastric and liver metabolism to overall zolpidem disposition and should clarify which specific Adh/Aldh isozymes are responsible for zolpidem metabolism. Such studies should also investigate whether first-pass metabolism is also related to sex differences in the pharmacological effects and side effects if zolpidem. Additionally, zolpidem (more likely the CYP-mediated hydroxyl metabolites) has been demonstrated to be a weak (*K*i = 122 uM) mechanism-based inhibitor (both time- and concentration-dependent) for CYP3A4, but due to the relatively high *K*i value, was deemed to be unlikely to cause clinical drug interactions (Polasek et al., 2010). Previous studies have only focused on pharmacodynamics interactions in the brain and not differences in ADH expression affecting bioavailability (Devaud and Morrow, 1994; Devaud et al., 1995; Tuk et al., 2002).

## Author Contributions

Designed study: CP, EF, and TS. Performed research: JS, SB, AL, and TS. Analyzed Data: CP and TS. Wrote Manuscript: CP, WT, and TS. Critical manuscript revision: CP, WT, TS, PS, EF, KC, RF, JP, AM, and MJ.

## Conflict of Interest Statement

The authors declare that the research was conducted in the absence of any commercial or financial relationships that could be construed as a potential conflict of interest.

The content of this publication does not necessarily reflect the views or policies of the Department of Health and Human Services, nor does mention of trade names, commercial products, or organizations imply endorsement by the U.S. Government.

## References

[B1] Aasmoe L. Aarbakke J. (1999). Sex-dependent induction of alcohol dehydrogenase activity in rats. *Biochem. Pharmacol.* 57 1067–1072. 10.1016/S0006-2952(99)00003-9 10796077

[B2] Bailer A. J. (1988). Testing for the equality of area under the curves when using destructive measurement techniques. *J. Pharmacokinet. Biopharm.* 16 303–309. 10.1007/BF01062139 3221328

[B3] Brien J. F. Peachey J. E. Rogers B. J. Loomis C. W. (1978). A study of the calcium carbimide-ethanol interaction in man. *Eur. J. Clin. Pharmacol.* 14 133–141. 10.1007/BF00607445 720375

[B4] Cederbaum A. I. (2012). Alcohol metabolism. *Clin. Liver Dis.* 16 667–685. 10.1016/j.cld.2012.08.002 23101976 PMC3484320

[B5] Chen J. Yanagawa Y. Yoshida A. (1996). Molecular mechanism of null expression of aldehyde dehydrogenase-1 in rat liver. *Biochem. Genet.* 34 109–116. 10.1007/BF02396244 8734411

[B6] Christoffersen B. Raun K. Svendsen O. Fledelius C. Golozoubova V. (2006). Evalution of the castrated male Sprague-Dawley rat as a model of the metabolic syndrome and type 2 diabetes. *Int. J. Obes. (Lond)* 30 1288–1297. 10.1038/sj.ijo.0803261 16505834

[B7] Ciccone G. K. Holdcroft A. (1999). Drugs and sex differences: a review of drugs relating to anaesthesia. *Br. J. Anaesth.* 82 255–265. 10.1093/bja/82.2.255 10365004

[B8] Crabb D. W. Bosron W. F. Li T. K. (1987). Ethanol metabolism. *Pharmacol. Ther.* 34 59–73. 10.1016/0163-7258(87)90092-1 3310044

[B9] Devaud L. L. Morrow A. L. (1994). Effects of chronic ethanol administration on [3H]zolpidem binding in rat brain. *Eur. J. Pharmacol.* 267 243–247. 10.1016/0922-4106(94)90177-5 8050485

[B10] Devaud L. L. Morrow A. L. Criswell H. E. Breese G. R. Duncan G. E. (1995). Regional differences in the effects of chronic ethanol administration on [3H]zolpidem binding in rat brain. *Alcohol. Clin. Exp. Res.* 19 910–914. 10.1111/j.1530-0277.1995.tb00966.x 7485838

[B11] Dulisch M. L. (1976). A castration procedure for the rabbit, rat, hamster, and guinea pig. *J. Zoo Anim. Med.* 7 8–11. 10.2307/20094380

[B12] Estonius M. Danielsson O. Karlsson C. Persson H. Jornvall H. Hoog J. O. (1993). Distribution of alcohol and sorbitol dehydrogenases. Assessment of mRNA species in mammalian tissues. *Eur. J. Biochem.* 215 497–503. 8344317 10.1111/j.1432-1033.1993.tb18059.x

[B13] Food and Drug Administration [FDA] (2008). *Ambien^®^ Prescribing Information*. Washington, DC: Food, and Drug Administration.

[B14] Food and Drug Administration [FDA] (2015). *Guidance for Industry: Analytical Procedures and Methods Validation for Drugs and Biologics*. Washington, DC: Food, and Drug Administration.

[B15] Frezza M. di Padova C. Pozzato G. Terpin M. Baraona E. Lieber C. S. (1990). High blood alcohol levels in women. The role of decreased gastric alcohol dehydrogenase activity and first-pass metabolism. *N. Engl. J. Med.* 322 95–99. 2248624 10.1056/NEJM199001113220205

[B16] Garrigou-Gadenne D. Burke J. T. Durand A. Depoortere H. Thenot J. P. Morselli P. L. (1989). Pharmacokinetics, brain distribution and pharmaco-electrocorticographic profile of zolpidem, a new hypnotic, in the rat. *J. Pharmacol. Exp. Ther.* 248 1283–1288. 2703975

[B17] Gessner P. A. Gessner T. (1992). Disulfiram and its Metabolite Diethyldithiocarbamate: Pharmacology and Status in the Treatment of Alcoholism, HIV Infections, AIDS and Heavy Metal Toxicity. London: Chapman, and Hall.

[B18] Gillet G. (1991) “In vitro and in vivo metabolism of zolpidem in three animal species and in man,” in *Proceedings of the Third International ISSX Meeting* Amsterdam.

[B19] Greenblatt D. J. Harmatz J. S. Roth T. Singh N. N. Moline M. L. Harris S. C. (2013). Comparison of pharmacokinetic profiles of zolpidem buffered sublingual tablet and zolpidem oral immediate-release tablet: results from a single-center, single-dose, randomized, open-label crossover study in healthy adults. *Clin. Ther.* 35 604–611. 10.1016/j.clinthera.2013.03.007 23541711

[B20] Greenblatt D. J. Harmatz J. S. Singh N. N. Steinberg F. Roth T. Moline M. L. (2014a). Gender differences in pharmacokinetics and pharmacodynamics of zolpidem following sublingual administration. *J. Clin. Pharmacol.* 54 282–290. 10.1002/jcph.220 24203450

[B21] Greenblatt D. J. Harmatz J. S. Singh N. N. Steinberg F. Roth T. Harris S. C. (2014b). Pharmacokinetics of zolpidem from sublingual zolpidem tartrate tablets in healthy elderly versus non-elderly subjects. *Drugs Aging* 31 731–736. 10.1007/s40266-014-0211-3 25246162

[B22] Greenblatt D. J. Harmatz J. S. von Moltke L. L. Wright C. E. Durol A. L. Harrel-Joseph L. M. (2000). Comparative kinetics and response to the benzodiazepine agonists triazolam and zolpidem: evaluation of sex-dependent differences. *J. Pharmacol. Exp. Ther.* 293 435–443. 10773013

[B23] Guo T. Mao G. Zhao L. Xia D. Yang L. (2014). Comparative pharmacokinetics of zolpidem tartrate in five ethnic populations of China. *Acta Pharm. Sin. B* 4 146–150. 10.1016/j.apsb.2014.02.001 26579377 PMC4590726

[B24] Hedberg J. J. Hoog J. O. Nilsson J. A. Xi Z. Elfwing A. Grafstrom R. C. (2000). Expression of alcohol dehydrogenase 3 in tissue and cultured cells from human oral mucosa. *Am. J. Pathol.* 157 1745–1755. 10.1016/S0002-9440(10)64811-0 11073833 PMC1885748

[B25] Keung W. M. Vallee B. L. (1993). Daidzin: a potent, selective inhibitor of human mitochondrial aldehyde dehydrogenase. *Proc. Natl. Acad. Sci. U.S.A.* 90 1247–1251. 10.1073/pnas.90.4.1247 8433985 PMC45849

[B26] Koppaka V. Thompson D. C. Chen Y. Ellermann M. Nicolaou K. C. Juvonen R. O. (2012). Aldehyde dehydrogenase inhibitors: a comprehensive review of the pharmacology, mechanism of action, substrate specificity, and clinical application. *Pharmacol. Rev.* 64 520–539. 10.1124/pr.111.005538 22544865 PMC3400832

[B27] Lindahl R. (1992). Aldehyde dehydrogenases and their role in carcinogenesis. *Crit. Rev. Biochem. Mol. Biol* 27 283–335. 10.3109/10409239209082565 1521460

[B28] Moreno A. Pares A. Ortiz J. Enriquez J. Pares X. (1994). Alcohol dehydrogenase from human stomach: variability in normal mucosa and effect of age, gender, ADH3 phenotype and gastric region. *Alcohol Alcohol.* 29 663–671. 7695781

[B29] Nedelman J. R. Jia X. (1998). An extension of Satterthwaite’s approximation applied to pharmacokinetics. *J. Biopharm. Stat.* 8 317–328. 10.1080/10543409808835241 9598425

[B30] Olubodun J. O. Ochs H. R. von Moltke L. L. Roubenoff R. Hesse L. M. Harmatz J. S. (2003). Pharmacokinetic properties of zolpidem in elderly and young adults: possible modulation by testosterone in men. *Br. J. Clin. Pharmacol.* 56 297–304. 10.1046/j.0306-5251.2003.01852.x 12919178 PMC1884349

[B31] Parlesak A. Billinger M. H. Bode C. Bode J. C. (2002). Gastric alcohol dehydrogenase activity in man: influence of gender, age, alcohol consumption and smoking in a caucasian population. *Alcohol Alcohol.* 37 388–393. 10.1093/alcalc/37.4.388 12107043

[B32] Pichard L. Gillet G. Bonfils C. Domergue J. Thenot J. P. Maurel P. (1995). Oxidative metabolism of zolpidem by human liver cytochrome P450S. *Drug Metab. Dispos.* 23 1253–1262. 8591727

[B33] Polasek T. M. Sadagopal J. S. Elliot D. J. Miners J. O. (2010). In vitro-in vivo extrapolation of zolpidem as a perpetrator of metabolic interactions involving CYP3A. *Eur. J. Clin. Pharmacol.* 66 275–283. 10.1007/s00228-009-0760-2 20012430

[B34] Quertemont E. (2004). Genetic polymorphism in ethanol metabolism: acetaldehyde contribution to alcohol abuse and alcoholism. *Mol. Psychiatry* 9 570–581. 10.1038/sj.mp.4001497 15164086

[B35] Sharkawi M. (1980). Inhibition of alcohol dehydrogenase by disulfiram; possible relation to the disulfiram-ethanol reaction. *Life Sci.* 27 1939–1945. 10.1016/0024-3205(80)90412-9 7010034

[B36] Stockmann C. Sherwin C. M. Buterbaugh W. Spigarelli M. G. Gottschlich M. M. Healy D. (2014). Preliminary assessment of zolpidem pharmacokinetics in pediatric burn patients. *Ther. Drug Monit.* 36 295–301. 10.1097/FTD.0000000000000017 24365985

[B37] Tuk B. van Gool T. Danhof M. (2002). Mechanism-based pharmacodynamic modeling of the interaction of midazolam, bretazenil, and zolpidem with ethanol. *J. Pharmacokinet. Pharmacodyn.* 29 235–250. 10.1023/A:1020202806759 12449497

[B38] Verster J. C. van de Loo A. J. Moline M. L. Roth T. (2014). Middle-of-the-night administration of sleep medication: a critical review of the effects on next morning driving ability. *Curr. Drug Saf.* 9 205–211. 10.2174/1574886309666140601210422 24909576

[B39] Visser S. A. Wolters F. L. van der Graaf P. H. Peletier L. A. Danhof M. (2003). Dose-dependent EEG effects of zolpidem provide evidence for GABA(A) receptor subtype selectivity in vivo. *J. Pharmacol. Exp. Ther.* 304 1251–1257. 10.1124/jpet.102.044859 12604703

[B40] Vlase L. Popa A. Neag M. Muntean D. Baldea I. Leucuta S. E. (2011). Pharmacokinetic interaction between zolpidem and carbamazepine in healthy volunteers. *J. Clin. Pharmacol.* 51 1233–1236. 10.1177/0091270010383690 21098143

[B41] Westerlund M. Belin A. C. Felder M. R. Olson L. Galter D. (2007). High and complementary expression patterns of alcohol and aldehyde dehydrogenases in the gastrointestinal tract: implications for Parkinson’s disease. *FEBS J.* 274 1212–1223. 10.1111/j.1742-4658.2007.05665.x 17257171

[B42] Wolbold R. Klein K. Burk O. Nussler A. K. Neuhaus P. Eichelbaum M. (2003). Sex is a major determinant of CYP3A4 expression in human liver. *Hepatology* 38 978–988. 10.1002/hep.1840380424 14512885

[B43] Xie Y. Q. Takimoto K. Pitot H. C. Miskimins W. K. Lindahl R. (1996). Characterization of the rat Class 3 aldehyde dehydrogenase gene promoter. *Nucleic Acids Res.* 24 4185–4191. 10.1093/nar/24.21.4185 8932370 PMC146258

[B44] Yoon I. S. Choi M. K. Kim J. S. Shim C. K. Chung S. J. Kim D. D. (2011). Pharmacokinetics and first-pass elimination of metoprolol in rats: contribution of intestinal first-pass extraction to low bioavailability of metoprolol. *Xenobiotica* 41 243–251. 10.3109/00498254.2010.538090 21128757

